# Single-port robotic cholecystectomy. Initial and pioneer experience in Brazil

**DOI:** 10.1590/S1679-45082015RC3275

**Published:** 2015

**Authors:** Vladimir Schraibman, Marina Gabrielle Epstein, Gabriel Naman Maccapani, Antônio Luiz de Vasconcellos Macedo

**Affiliations:** 1 Hospital Israelita Albert Einstein, São Paulo, SP, Brazil.

**Keywords:** Cholecystectomy, Cholecystectomy/methods, Minimally invasive surgical procedures, Gallbladder, Gallbladder diseases

## Abstract

The technique of a single-port laparoscopy was developed over the last years as an attempt to lower surgical aggression and improve the aesthetic results of the minimally invasive surgery. A new robotic platform used with the da Vinci^®^ Robotic System Single-Site System^®^ (Intuitive Surgical, Sunnyvale, California, United States) was recently launched on the global market and is still not documented in Brazil. The authors report on the first four robotic single-port cholecystectomies performed with this da Vinci^®^ Robotic System in Brazil.

## INTRODUCTION

Laparoscopic cholecystectomy, initially introduced in 1986, was the major landmark of minimally invasive surgery in the area of digestive tract surgery over the last years. Currently, laparoscopic cholecystectomy is the gold standard surgery in treating cholecystopathies,^(1)^ in addition to diseases with indication for gallbladder removal.

On the other hand, SILS – single-incision laparoscopic surgery – is a recent technological advance in minimally invasive surgery. It was developed as a less invasive alternative to conventional laparoscopy.^(2)^ It uses a single 2 to 2.5cm incision for its performance, without the need for multiple punctures. The first laparoscopic cholecystectomy by single port was done by Navarra, in 1997.^(3)^ Despite the promising results published in literature, there were significant hindrances reported for this approach, such as difficulty in pulling the gallbladder, in maintaining the dissection precise and delicate, and maintaining critical visibility.^(4,5)^ Other studies also reported a greater incidence of biliary tract lesions in these procedures, due to the great difficulty in dissecting the cystic duct and artery because of collision of forceps.^(4,5)^ Consequently, the acceptance and use of SILS are still controversial.

In this way, over the last five years, the Robotic da Vinci^®^ Single-Site System^®^ (Intuitive Surgical, Sunnyvale, California, United States), was developed. In it, there is inversion of the instruments with no need for great effort on the part of the surgeon, allowing wider movements and better ergonomics, when compared to non-robotic single-port laparoscopic operations.^(1)^ In addition to three-dimension visualization, the position of the surgeon sitting next to the patient, at the robot console, and the precision in dissection of the anatomical structures, allow a more precise surgery, without forceps collisions. The initial studies demonstrated that this technique is safe and effective, and can help resolve the technical limitations found in laparoscopy.^(1,2) ^Robotic surgery presents a stable perspective, arm movements linked by computerized inversion, and instruments that allow a high degree of freedom.^(1,5,6)^ This study shows the first four cases of surgery using the robotic Single-Site System for gallbladder removal.

## METHODS

On July 9, 2014, in an unprecedented way in Brazil, four patients were submitted to single-port cholecystectomy using the platform da Vinci^®^ Single-Site. The patients were operated on by two surgeons, ALVM and VS, two cases each, at *Hospital Israelita Albert Einstein*.

The demographic data of patients included age, body mass index, prior operations, and clinical diagnosis.

The data analyzed were operative time, skin incision, trochanter placement, docking (coupling time between the robot and the patient), time on the console, and length of hospital stay.

### Surgical technique

**Figure 1:**
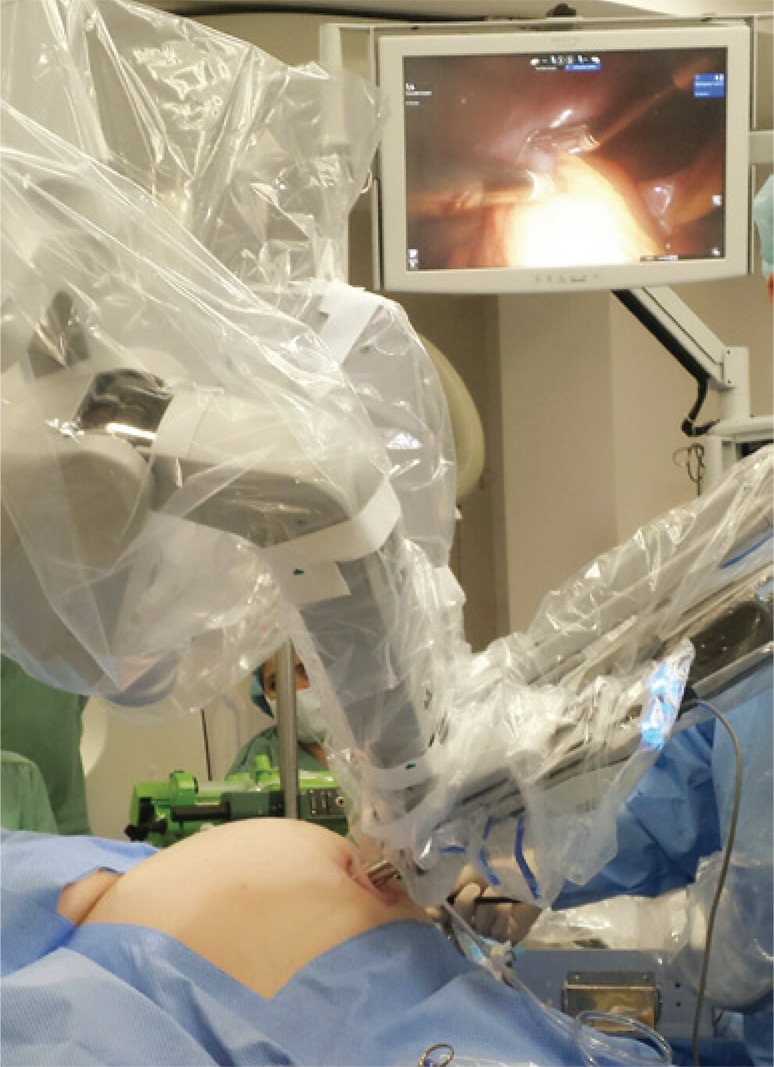
Position of the robot, port, and assistant during the surgery

**Figure 2:**
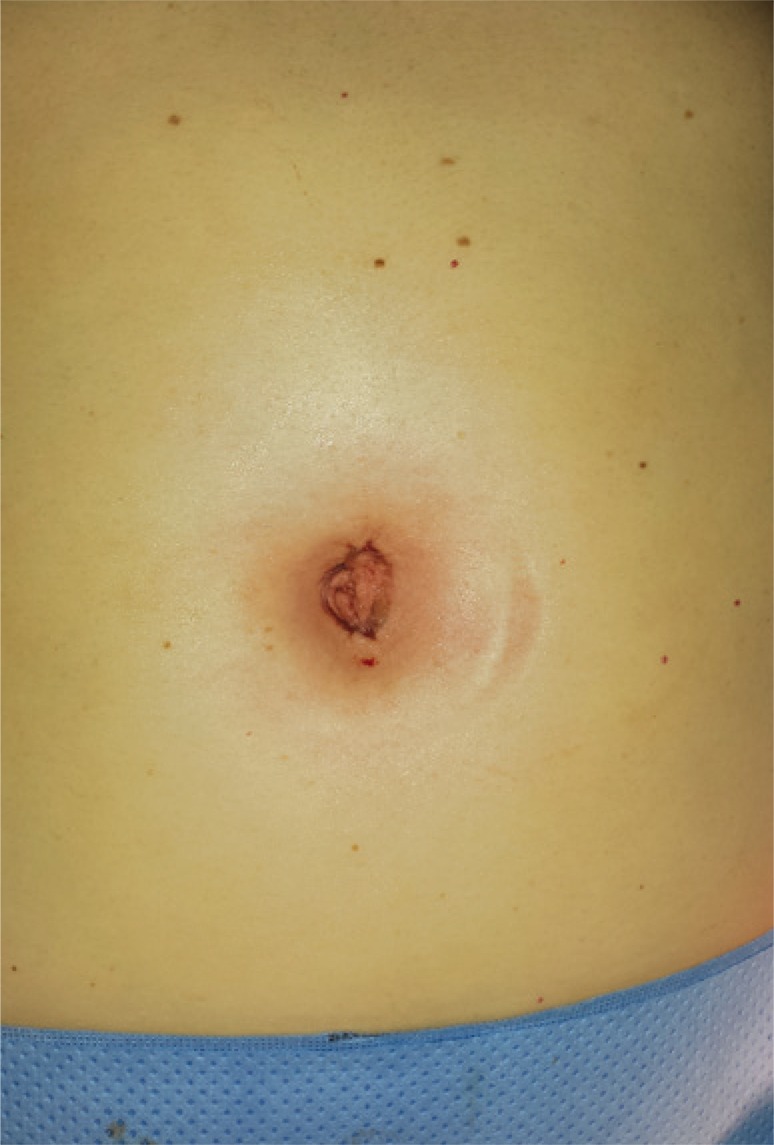
Final aspect of the abdomen after closing of the single orifice

Single-port robotic cholecystectomy is performed using the da Vinci^®^ Single-Site. The patient is placed in supine position under general anesthesia. A 2.5cm umbilical incision is made with dissection to the peritoneal cavity. The da Vinci^®^ Single-Site port is placed and pneumoperitoneum is initiated. The patient is placed in an inclined position with slight left lateral decubitus. The robot is place over the patient’s right shoulder. After introduction of the camera, the trochanters are placed under direct view. Next, docking (coupling with the robot) takes place and the fundus of the gallbladder is pulled towards the patient’s right shoulder by the assistant (Figure 1). The triangle of Calot is exposed. Dissection is performed with the robotic hook-type forceps and Maryland-type robotic forceps. After identification of the cystic duct and the cystic artery, both structures are clipped with the Hem-o-lock^® ^(Teleflex Medical, Ireland) and sectioned with scissors. The gallbladder is detached from the hepatic bed with the robotic hook. Hemostasis is conducted with bipolar forceps or robotic hook. The robotic instruments and the camera are removed, and the robot is disengaged. The piece is removed together with the single-port, after review of hemostasis of the hepatic bed. The wound is closed by layers and the skin in sutured with intradermal stitches (Figure 2).

## RESULTS

All cases were concluded without complications with the da Vinci^® ^SI platform. All patients presented with cholelithiasis as diagnosis for surgical indication, and none of them presented with acute cholecystitis or signs of gallbladder tumor.

In 50% of cases, patients had prior operations, and the greatest difficulty was in the adhesions around the gallbladder. In these cases, we noted a prolonged time on the console, due to the difficulty in detaching adhesions with the robotic single-port forceps.

There were no complications, such as biliary tract lesion, bleeding, or need for reoperation.

### Case 1

ESP, 72-year-old male, acute biliary pancreatitis, resolved.

Discharged on the first postoperative day.

### Case 2

DMFSM, 67-year-old female, diabetic, obese, prior hiatal hernioplasty. Discharged on the first postoperative day.

### Case 3

EEB, 49- year-old male, overweight. Discharged on the first postoperative day.

### Case 4

DC, 39 year-old male, overweight. Discharged on the first postoperative day.

All procedures were concluded with the robotic single-port device.

The demographic data of patients are summarized on table 1.

**Table 1:** Demographic data

Variables	Values
Age (mean)	56.7
Male (%)	75
BMI (kg/m^2^), mean	27.0
Prior abdominal surgery	2

BMI: body mass index.

Operative results are shown on table 2.

**Table 2:** Operative data

Variables	Values
Time of anesthesia (minute), mean	116
Docking (minute)	32.5
Operative time on console (minute), mean	39.7
Length of hospital stay (hour), mean	17

## DISCUSSION

Laparoscopic cholecystectomy has been performed for more than 20 years and it represents the gold standard for treatment of cholelithiasis all over the world. During the last few years, minimally invasive technology took an important step in its evolution, enabling, besides conventional laparoscopic surgery, the operation with a single port. Since its introduction, SILS has not been used as routine, primarily due to physical limitations of this technology, which compromises triangulation, ergonomics of the procedure, and quality of the view, leading to difficulty in exposing the anatomical structures and to increased risk of lesions to the biliary tract.^(5)^

The introduction of robotic cholecystectomy by a single port set a new landmark for SILS. The curved trochanters of the robotic system were designed to decrease the problem of triangulation and the single-port with entrance for the auxiliary forceps was made for traction of the gallbladder, with no need for percutaneous sutures.^(7)^

However, in a systematic review by Antoniou et al.,^(8)^ 29 studies of non-robotic SILS cholecystectomies were reviewed, including 1,166 patients, and a significant increase in the mean rate of complications was identified in cases where the gallbladder was resected, as compared to the mean complication rates reported in videolaparoscopic cholecystectomy.

In this way, the search began for technologies that might allow the operation by a single orifice with safety, precision, and low rate of complications. Initially introduced in the United States about three years ago, the robotic da Vinci^®^ Single-Site platform was introduced in Brazil, in 2014. In this study, we present the first four cases of single-port robotic cholecystectomy performed in Brazil. According to the initial results, the procedure seems safe and viable, having been concluded without conversions and with no postoperative complications.

The advantages of the robotic single port approach are better esthetic results, less postoperative pain due to the small single incision, visualization of the anatomical structures in three dimensions, stability of the instruments by the robotic platform, precision in dissections, and greater ease for the surgeon in concluding dissections made difficult by the single port. Additionally, the curved robotic semi-rigid instruments provide a safe platform for the performance of the procedures and overcome restrictions and limitations, when compared to laparoscopy via a single port.^(1,5)^ Despite the fact that this study presents a small number of patients operated on, it demonstrates the feasibility of the method and indicates the future possibility of adopting this method as gold standard in elective cholecystectomy, as is described in large reference centers for advanced surgery in the world.^(5,6)^

## CONCLUSION

Single-port robotic surgery is feasible and safe when performed by surgeons with prior experience in robotic surgery. Studies with large series of cases are necessary to establish the superiority of the single-port robotic procedure as compared to the single-incision laparoscopic surgery and videolaparoscopic cholecystectomy.

## References

[1] Vidovszky TJ, Carr AD, Farinholt GN, Ho HS, Smith WH, Ali MR (2014). Single-site robotic cholecystectomy in a broadly inclusive patient population: a prospective study. Ann Surg.

[2] Wren SM, Curet MJ (2011). Single-port robotic cholecystectomy: results from a first human use clinical study of the new da Vinci single-site surgical platform. Arch Surg.

[3] Navarra G, Pozza E, Occhionorelli S, Carcoforo P, Donini I (1997). One wound laparoscopic cholecystectomy. Br J Surg.

[4] Ruurda JP, van Vroonhoven TJ, Broeders IA (2002). Robot-assisted surgical systems: a new era in laparoscopic surgery. Ann R Coll Surg Engl.

[5] Uras C, Böler DE, Ergüner I, Hamzaoğlu I (2014). Robotic single port cholecystectomy (R-LESS-C): experience in 36 patients. Asian J Surg.

[6] Ayloo S, Choudhury N (2014). Single-site robotic cholecystectomy. JSLS.

[7] Pietrabissa A, Sbrana F, Morelli L, Badessi F, Pugliese L, Vinci A (2012). Overcoming the challenges of single-incision cholecystectomy with robotic single-site technology. Arch Surg.

[8] Antoniou SA, Pointner R, Granderath FA (2011). Single-incision laparoscopic cholecystectomy: a systematic review. Surg Endosc.

