# Supplementary material for: Single-port robotic cholecystectomy. Initial and pioneer experience in Brazil
Source: Einstein (Sao Paulo). 2015 Oct-Dec;13(4):607–10. doi: 10.1590/S1679-45082015RC3275 (PMC4878639; doi:10.1590/S1679-45082015RC3275)
Supplement: Translation

# Translation: Colecistectomia robótica por portal único. Experiência inicial e pioneira no Brasil

**Authors:** Vladimir Schraibman, Marina Gabrielle Epstein, Gabriel Naman Maccapani, Antônio Luiz de Vasconcellos Macedo

**Affiliations:** 1 Hospital Israelita Albert Einstein, São Paulo, SP, Brazil.

**Keywords:** Colecistectomia, Colecistectomia/métodos, Procedimentos cirúrgicos minimamente invasivos, Vesícula biliar, Doenças da vesícula biliar

## Abstract

A técnica de um único portal laparoscópico foi desenvolvida ao longo dos últimos anos, numa tentativa de diminuir a agressão cirúrgica e melhorar ainda mais os resultados estéticos da cirurgia minimamente invasiva. Uma nova plataforma robótica, usada com o Sistema Robótico da Vinci^®^ *Single-Site System* ^*®*^ (*Intuitive Surgical*, Sunnyvale, Califórnia, Estados Unidos), foi recentemente lançada no mercado mundial e é ainda inédita no Brasil. Os autores relatam as primeiras quatro colecistectomias robóticas com portal único realizadas com este Sistema Robótico da Vinci^®^ no Brasil.

## INTRODUÇÃO

A colecistectomia laparoscópica inicialmente introduzida em 1986 foi o principal marco da cirurgia minimamente invasiva na área de cirurgia do aparelho digestivo nos últimos anos. Atualmente, a colecistectomia laparoscópica é a cirurgia padrão-ouro no tratamento das colecistopatias,^(1)^ além de doenças com indicação de remoção da vesícula biliar.

Por outro lado, a cirurgia de incisão única laparoscópica (single-incision laparoscopic surgery - SILS) é um recente avanço tecnológico em cirurgia minimamente invasiva. Foi desenvolvida como uma alternativa menos invasiva à laparoscopia convencional.^(2)^ Trata-se de uma incisão única de cerca de 2 a 2,5cm para a realização de cirurgias laparoscópicas sem a necessidade de se realizarem punções múltiplas. A primeira colecistectomia laparoscópica por portal único foi feita por Navarra, em 1997.^(3)^ Apesar dos resultados promissores publicados na literatura, foram relatadas dificuldades significativas dessa abordagem, como dificuldade para tracionar a vesícula biliar, dificuldade em se manter a dissecção precisa e delicada, e a manutenção da visão crítica.^(4,5)^ Outros trabalhos ainda relatam a maior incidência de lesão da via biliar nesses procedimentos, devido à grande dificuldade em se dissecarem o ducto e a artéria cística, oriunda da colisão das pinças.^(4,5)^ Como consequência, a aceitação e o uso da SILS permanecem controversos.

Desse modo, foi desenvolvido, nos últimos 5 anos, o Sistema Robótico da Vinci^®^ *Single-Site System* ^®^ (*Intuitive Surgical*, Sunnyvale, Califórnia, Estados Unidos). Nele, ocorre a inversão dos instrumentos sem necessidade de grande esforço por parte do cirurgião, permitindo movimentos mais amplos e uma melhor ergonomia, quando comparado às cirurgias laparoscópicas não robóticas, de portal único.^(1)^ Além da visualização tridimensional, o posicionamento do cirurgião, ao lado do paciente, no console do robô, e a precisão na dissecção das estruturas anatômicas permitem uma cirurgia mais precisa e sem colisões de pinças. Estudos iniciais demonstraram que essa técnica é segura e eficaz, podendo ajudar a resolver as limitações técnicas encontradas na laparoscopia.^(1,2) ^A cirurgia robótica apresenta uma ótica estável, movimentos dos braços ligados por inversão informatizada e instrumentos que proporcionam um elevado grau de liberdade.^(1,5,6)^ Este trabalho apresenta os quatro primeiros casos de cirurgia usando o *Single-SiteSystem* robótico para remoção da vesícula biliar.

## MÉTODOS

Em 9/7/2014, de modo inédito no Brasil, quatro pacientes foram submetidos à colecistectomia por portal único, com a plataforma da Vinci^®^ *Single-Site*. Os casos foram operados por dois cirurgiões, ALVM e VS, dois casos cada um, no Hospital Israelita Albert Einstein.

Os dados demográficos dos pacientes considerados foram idade, índice de massa corporal, cirurgias anteriores e diagnóstico clínico.

Os dados analisados foram: tempo operatório, incisão da pele, colocação de trocânteres, *docking* (tempo de acoplamento do robô ao paciente), tempo no console e tempo de internação.

### Técnica cirúrgica

A colecistectomia robótica por portal único é realizada com o da Vinci^®^ *Single-Site*. O paciente é colocado em posição supina sob anestesia geral. É feita uma incisão umbilical de 2,5cm e realizada a dissecção até a cavidade peritoneal. O portal da Vinci^®^ *Single-Site* é colocado e o pneumoperitônio é iniciado. O paciente é posicionado em proclive e com um leve decúbito lateral esquerdo. O robô é colocado sobre o ombro direito do paciente. Após a introdução da câmera, os trocânteres são colocados sob visão direta. É realizado o *docking* (acoplamento do robô), e o fundo da vesícula biliar é tracionado em direção ao ombro direito do paciente pelo assistente (Figura 1). O triângulo de Calot é exposto. A dissecção é realizada com pinça robótica tipo gancho e uma pinça robótica tipo Maryland. Após a identificação do ducto cístico e da artéria cística, ambas as estruturas são clipadas com Hem-o-lock^®^ (*Teleflex Medical*, Irlanda) e seccionados com tesoura. A vesícula biliar é descolada do leito hepático com o gancho robótico. A hemostasia é realizada com pinça bipolar ou gancho robótico. Os instrumentos robóticos e a câmera são retirados, e o robô é desencaixado. A retirada da peça é feita em conjunto com a retirada do portal único, após a revisão da hemostasia do leito hepático. Realiza-se o fechamento por planos, e é feita a síntese da pele com pontos intradérmicos (Figura 2).

**Figura 1:** Disposição do robô, portal e assistente durante a cirurgia

**Figura 2:** Aspecto final do abdômen apos fechamento do orifício único

## RESULTADOS

Todos os casos foram concluídos sem intercorrências com a plataforma da Vinci^®^ SI. Todos os pacientes apresentavam colelitíase como diagnóstico de indicação cirúrgica, e nenhum deles apresentava colecistite aguda ou indícios de tumor de vesícula biliar.

Em 50% dos casos, os pacientes tinham cirurgia prévia, e a dificuldade maior consistiu nas aderências ao redor da vesícula biliar. Nesses casos, observamos um tempo prolongado no console, devido à dificuldade da lise de aderências com as pinças do *single port* robótico.

Nenhum caso teve qualquer tipo de complicação, como lesão de via biliar, sangramento ou necessidade de reabordagem cirúrgica.

### Caso 1

ESP, 72 anos, sexo masculino, pancreatite aguda biliar resolvida.

Alta no primeiro pós-operatório.

### Caso 2

DMFSM, 67 anos, sexo feminino, diabética, obesa, hernioplastia hiatal prévia. Alta no primeiro pós-operatório.

### Caso 3

EEB, 49 anos, sexo masculino, sobrepeso. Alta no primeiro pós-operatório.

### Caso 4

DC, 39 anos, sexo masculino, sobrepeso. Alta no primeiro pós-operatório.

Todos os procedimentos foram concluídos com o portal único robótico.

Os dados demográficos dos pacientes estão resumidos na tabela 1.

**Tabela 1:** Dados demográficos

Variáveis	Valores
Idade (média)	56,7
Sexo masculino (%)	75
IMC (kg/m^2^), média	27,0
Cirurgia prévia abdominal	2
IMC: índice de massa corporal.	

Os resultados operatórios são mostrados na tabela 2.

**Tabela 2:** Dados operatórios

Variáveis	Valores
Tempo de anestesia (minuto), média	116
*Docking* (minuto)	32,5
Tempo cirúrgico no console (minuto), média	39,7
Tempo de internação (hora), média	17

## DISCUSSÃO

A colecistectomia laparoscópica tem sido realizada há mais de 20 anos e representa o tratamento padrão-ouro para a colelitíase em todo o mundo. Durante os últimos anos, a tecnologia minimamente invasiva deu um passo importante em sua evolução, possibilitando, além da cirurgia laparoscópica convencional, a cirurgia com portal único. Desde sua introdução, a SILS não foi realizada de rotina, principalmente devido a limitações físicas dessa tecnologia, que comprometem triangulação, ergonomia do procedimento e a qualidade de visão, levando à dificuldade de exposição das estruturas anatômicas e ao risco aumentado de lesão da via biliar.^(5)^

A introdução da colecistectomia robótica por portal único criou um novo marco para SILS. Os trocânteres curvos do sistema robótico foram desenhados para diminuir o problema da triangulação e o portal único com entrada para pinça de auxílio foi feito para tração da vesícula biliar, sem necessidade de suturas percutâneas.^(7)^

Porém, em uma revisão sistemática de Antoniou et al.,^(8)^ foram analisados 29 estudos de colecistectomias SILS não robóticas, incluindo 1.166 pacientes, e foi identificado um aumento significativo da taxa média de complicações nos casos em que foram realizadas ressecção da vesícula biliar, em relação às médias históricas de complicações de colecistectomia videolaparoscópicas.

Desse modo, deu-se início à procura de tecnologias que pudessem permitir a realização de cirurgia por orifício único com segurança, precisão e com baixo índice de complicações. Inicialmente introduzida nos Estados Unidos, há cerca de 3 anos, a plataforma robótica da Vinci^®^ *Single-Site* foi introduzida no Brasil em 2014. Apresentamos, neste trabalho, os quatro primeiros casos de colecistectomia robótica por portal único realizados no Brasil. Pelos resultados iniciais, o procedimento parece ser seguro e factível, sendo concluído sem conversões e sem complicações no pós-operatório.

As vantagens do portal único robótico são melhores resultados estéticos, diminuição da dor pós-operatória devido à pequena incisão única, visualização das estruturas anatômicas em três dimensões, estabilidade dos instrumentos pela plataforma robótica, precisão nas dissecções e maior facilidade ao cirurgião em concluir dissecções dificultadas pelo portal único. Além disso, os instrumentos semirrígidos curvos robóticos fornecem uma plataforma segura para a realização dos procedimentos e superam as restrições e limitações, quando comparados à laparoscopia por portal único.^(1,5)^ Apesar deste trabalho apresentar ainda um pequeno número de pacientes operados, ele demonstra a factibilidade do método e indica a possibilidade futura de adotar essa técnica como padrão-ouro em colecistectomia eletiva, assim como descrito em grandes centros de referência em cirurgia avançada no mundo.^(5,6)^

## CONCLUSÃO

A cirurgia robótica por portal único é factível e segura, quando realizada por cirurgiões com experiência em cirurgia robótica prévia. Estudos com grandes séries são necessários, a fim de se estabelecer a superioridade do *single port* robótico frente a *single-incision laparoscopic surgery* e a colecistectomia videolaparoscópica.
